# New York Odontological Society

**Published:** 1893-05

**Authors:** 

**Affiliations:** New York Odontological Society


### NEW YORK ODONTOLOGICAL SOCIETY.

A REGULAR meeting of the New York Odontological Society was held on Tuesday evening, February 21, 1893, at the New York Academy of Medicine, No. 17 West Forty-third Street, New York City, the President, Dr. Woodward, occupying the chair.

The minutes of the previous meeting were read and approved.

### INCIDENTS OF OFFICE PRACTICE.

*Dr. Brewster.*—I met with a little accident the other day which I would like to relate. I had been treating a gentleman about fifty years of age, whose lower left first molar was affected with periodontitis. A sac was formed after about six days, and I found it necessary to evacuate the cavity. I did so with full knowledge of what I was about to do. The lance entered properly, and I noticed immediately the spurting of arterial blood. I had taken every precaution not to come in contact with the artery. I let it go long enough to have the sac thoroughly washed out, and then I put pressure upon it to form a clot, and I had no further difficulty. Subsequently I made an examination, and found that the artery seemed very much nearer the bone than I had ever seen it. He was a very fleshy man. It may have occurred to many here, but this was the first time it had happened to me. I took especial care, too, that the edge of the lance should be towards the root.

*Dr. Brockway.*—I have been much impressed with the value of nitrate of silver in the treatment of caries of the teeth, as set forth in the papers of Dr. Stebbins, read before this and other societies; and I regard the ideas thus presented as among the most valuable that have ever been given to the profession.

Since the first of these papers was published—now nearly two years ago—I have made use of the method described in many cases with most gratifying results, and I think no one who saw the two little patients which Dr. Stebbins brought to one of our meetings, as exhibiting his treatment, could fail to be favorably impressed by the condition shown.

In this connection I wish to call attention to and commend a method of making application of nitrate of silver to cavities in the teeth employed by Dr. A. M. Holmes, and described by him in one of the recent journals.

The cavity being dried, a piece of gutta-percha is warmed and touched to some powdered crystals of the salt, then packed into the cavity, thus holding the caustic in contact with the surface to be acted on. This I have found to be a very simple and effective way of securing the desired result.

Doubtless many others present have made use of the treatment in question, and their experience would be of interest at this time.

*Dr. Howe.*—At the time Dr. Stebbins read his paper here I stated that in the summer of 1891 I had seen some of the cases he had treated, and that I had begun to treat cavities of decay in children's teeth with nitrate of silver immediately on my return in the autumn of that year. I would say now that I have seen within a few weeks some of the cases first treated,—more than eighteen months ago,—and found them in a very satisfactory condition. I

have come to regard this method of arresting decay as one of the most valuable additions to our armamentarium.

*Dr. S. E. Davenport.*—Nitrate of silver has been of considerable assistance to me, for although I have used it in but a small number of cases, in every one it has been satisfactory. For one little girl, whose teeth were quite a problem, by its use I have been able to check, without filling, the places which were most annoying, and which seemed to prove that the teeth were of very low structure. Two applications have sufficed to prevent the decay from spreading, and have taken from those teeth the extreme sensitiveness which was very troublesome before. In this little mouth there were decayed spots on the lingual side of all the sixth-year molars, as well as on the buccal surfaces, and the good results of the use of the nitrate of silver in those shallow, sensitive places causes me to think that the remedy has great value.

The president then introduced Dr. Dwight M. Clapp, of Boston, who spoke on the subject of "Combination Fillings," as follows:

*Dr. Clapp.*—It is now a little over four years since I read before this Society a short paper on combination fillings. That paper dealt exclusively with the combination of gold and amalgam. Within a few days I have looked over that paper again, and I find that, were I to rewrite it, I could not improve it, and to my mind the combination of gold and amalgam in the cases where it is indicated is unrivalled as a filling for the preservation of teeth. In the *Dental Cosmos* of July, 1892, there is an article dealing with various combinations, by Professor Miller, of Berlin. He states there, in regard to the joint filling of gold and amalgam, that its principal disadvantage is that there is no union between the two. Of course he refers to the old method of using gold and amalgam by first inserting the amalgam, allowing it to harden, and then forming a cavity in which gold is placed. A little later he refers to the article of which I have spoken, which I read before this Society, and he says that the accounts of the result of this work that have come to hand are very meagre, although satisfactory. I wish now to make a public expression of my own views and experience with this combination, and say that I regard it as a success, and that it is and will be one of our greatest helps. In fact, in the five years that I have used this method of filling, I have not found what could be termed a failure. I must admit that there has been a slight recurrence of decay in a few instances, but that was in cases where one might suspect faulty manipulation, and others that were very far gone to begin with.

I wish to say a few words in furtherance of combination fillings, and with some slight addition to what I read before you some four years ago.

Thinking that seeing is believing, I have prepared quite a number of specimens, and I hope they will do most of the talking.

The first specimen that I wish you to look at is a combination filling in the central, and with it is a cavity as nearly like the one that has been filled as I could make it. I would ask you to take particular notice of this specimen, because I shall refer to it later. In addition to gold and amalgam, I use cement very largely. Of course what I have to say to-night is not new to you. You have all used it probably in the same way that I do, but I would like to say one thing in regard to the successes and failures. I said the fillings, in my hands, for the last five years have been successes, but I have met with one great failure. It is this: although in the societies to which I belong I have talked combination fillings in season and out of season, yet I believe not one in ten uses combination fillings, and that not two per cent. of the practitioners know anything about the advantages and the great helps that come from them. In large cavities if we can so put in fillings that they make one mass with the tooth, provided the filling-materials are of the proper kind, it seems to me we are giving a better filling than if we put in something that is simply held in by shape. For instance, if you pile up a pier of bricks, you have simply a pile of bricks; but if you pile up a pier in which each brick is cemented to the other, you have more than a pile of bricks,—you have something that is solid and will hold. Consequently if we place in the tooth something that is cemented to it and held together all through, we have that which strengthens the tooth rather than weakens it. Do not misunderstand me. I do not mean that I place fillings in the tooth and depend entirely on cement for holding them in. I have been reported as doing that, with the meaning that I could not put them in any other way.

Another thing I wish to speak of in regard to this specimen is this: I made one like it and exhibited it to the American Academy in Boston, and I stated that the filling was put in with cement and gold, and it was finished with pieces of foil that I had picked up around the office,—meaning that it was gold that had been discarded for daily use. Not long since I was amused to read in the paper that "Dr. Clapp generally finishes with gold that he picks up around the office." However, I suppose we must expect little mistakes of this kind.

*Dr. Francis.*—When Dr. Clapp uses the term "cement," does he mean amalgam?

*Dr. Clapp.*—No; I mean oxyphosphate. My method is to prepare a cavity and place in it some cement. While the cement is soft, I put into it the filling-material, whatever it may be, gold or amalgam. In this specimen that I have here there is represented a dead molar with a very large cavity, involving the crown and the surface of the tooth clear to the gum. In this case, after the cavity is prepared, I would place in soft cement, and into the mesial portion of the cavity a little amalgam. In a minute or so, when the cement has hardened somewhat, I remove with an excavator the surplus cement at the edge of the cavity, so I have a cavity the whole opening of which is sure to be free from any cement. Then I place on the matrix and fill with amalgam, finishing with gold, or with amalgam alone, or with gold alone, as the case may be. The reason I have for putting the amalgam into the soft cement before adjusting the matrix is, that in most cases the opening to cavities is so small that if you adjust the matrix first, then place in the soft cement, and then the amalgam, you crowd the cement to the surface, and you cannot see to trim it off so as to be sure that none of the cement will come to the edge of the filling. In this particular case, I should now proceed with amalgam, and then with gold enough to cover it; then some more soft cement in the crown, and into that some gold, and when that had hardened I should weld the entire mass with gold. Then we have practically a pier built up with cement from top to bottom. The filling is a shell of metal, and to my mind the least metal put into the cavity, provided there is enough for strength, the better the filling.

The next specimen is where the cavity is somewhat like the one just spoken of, but in place of putting amalgam into the cement, I have used gold entirely. With this filling as it is now, I should adjust the matrix and finish the filling with gold, or perhaps with tin and gold.

I have here a sample of the filling of which I have just spoken, of cement and amalgam and gold. The cement was first placed, and a little amalgam inserted into that while it was soft; then the matrix was adjusted, a little more amalgam added, then sufficient gold to cover the amalgam, then more soft cement put into the crown, and gold into that, just enough of crystal mat gold to make a surface to build upon. It has not been consolidated. As soon as the cement hardens, mallet this down and finish with any cohesive gold.

Another class of cavities where I find it valuable to use cement is in the front teeth, especially in young teeth. We often find quite extensive cavities in the centrals, laterals, and cuspids in patients from eighteen to twenty years old. To fill with gold those cases where the teeth are of poor quality is somewhat disheartening, because we have all seen a few years later discolorations around these fillings, and they are not very satisfactory. In these cases I use cement, and while it is soft add the mat gold, and a minute after remove the surplus where it has oozed out, and then finish with gold. I formerly filled these by inserting the cement, letting it harden, making a cavity, and filling it as we would a simple cavity with gold. I find it much more satisfactory to put the gold into the soft cement, thereby uniting tooth and filling.

Here is a specimen of a completed filling. It shows amalgam at the cervical wall and is finished with gold. This is one of those cases such as we often find where decay has gone so far that when it is removed the cavity is in such a shape that it would not hold a filling, and to make it retain the filling we would either expose the nerve or very much weaken the tooth. You know that in a bicuspid, if we cut down to square the cervical portion of the cavity, we cut into the tooth where it is the weakest, and where teeth are so often broken. With this method of using cement, we simply have to take out decay, relying on the cement, *at this portion*, to hold in the filling. I do not say that I can rely wholly on the cement for retaining the entire filling; I think it is necessary to have, somewhere in the cavity, retaining-points that will assist in securing it; what I am trying to find out is how much reliance we can place on cement for holding in fillings.

Here is a specimen which will illustrate thoroughly, I think, the advantage of amalgam for the cervical portion of the filling. It is the specimen that has the matrix adjusted. It is nearly a shell. In many cases it perhaps would not do to fill a cavity of this kind with the weak walls that this cavity has, because if a very strong masticating force were brought upon it, it might fracture the walls; but in many cases a tooth no stronger than this can be made serviceable for many years. Often these give us much trouble. This is a distal cavity in a superior or inferior molar; the decay has gone below the gum, and has extended out laterally towards the tongue and cheek, and, what is more, it is a cavity where it is impossible to use the rubber-dam and make it stay below the margin. In these cases I have been in the habit of using the matrix first. We can carry the thin matrix down below the decay, even down until we touch the alveola. Then the rubber can be adjusted over the matrix and the excavation finished. Of course, in these cases it is necessary to do most of the excavating before placing on the matrix, because we should not be able to see with it on. It is in this class of cases that amalgam is peculiarly serviceable. It can be packed against the matrix, leaving the main portion of the cavity open, into which cement can be placed and then finish with amalgam.

Tin-gold and soft foil and tin are remarkably good filling-materials, but, good as they are, you cannot use them in places that are inaccessible, where amalgam many times can be packed.

I have here several specimens in one. In the first place, here are two crown cavities with strong enamel walls, but very much hollowed out underneath. Those cavities might be filled with gold entirely, but it would be a long and tedious operation, and not particularly satisfactory when completed. Last Saturday I removed a crown filling something after this kind. Decay had extended in all directions. The filling was evidently carefully put in, but the shape of the cavity prevented a perfect packing of the gold. In these cavities I should put in cement. Here is another advantage of the cement. It is not necessary to waste tooth-substance in shaping a cavity. After the decay is removed, in most cases of this kind, no more cutting is necessary. Here is another case where a tooth is badly broken down, and it did not seem advisable to make a contour filling, and in it has been placed some cement and a little amalgam. The filling will be finished with amalgam. Here is another crown cavity where the cement has been put in and mat gold packed into it. Here is a cavity of gold and amalgam, which is partially completed. Here is a completed filling of gold and amalgam. In this special case it happened to be an upper molar, and it was so situated in the mouth that if amalgam had been placed in the bottom of the cavity, and the cavity had been filled one-third or one-half full, it would have shown from the outside; when the person opened his mouth, the black would have been unsightly. In this case amalgam has only been put on the lingual portion of the cavity, and the gold has been carried clear to the angle.

Tin-gold is a combination that has been used very extensively by certain operators. The first one to introduce it, I think, was Dr. Abbott, of Berlin, many years ago. To my mind it is a very valuable material. It is invaluable in many cavities. At the cervical wall of cavities, in place of amalgam, where a combination can be made of gold and tin-gold, it makes a most excellent filling. I am using that now, in many cases, where I formerly used amalgam with gold. Cases that are difficult to fill in a very permanent manner are small pin-head cavities between bicuspids and molars, and between bicuspids. We often find a case where the mesial side of a sixth-year molar is decayed, and the cavity must be cut down from the crown. Next to that on the distal surface of the bicuspid is a small pin-head cavity. In those cases tin-gold is a remarkably good filling. In many buccal cavities of wisdom-teeth and molars it is excellent, and also in crown cavities of sixth-year molars. This specimen has four fillings in it,—two crown cavities, one approximal, and one buccal. I have had occasion to examine many of these fillings after a few months, and they have become quite hard. One advantage over amalgam is the greater malleability, and they never turn the tooth dark. The tooth is never stained from these fillings, although the fillings themselves turn dark.

In this specimen that I mentioned first, you notice that the cavity in the central that is not filled has a slight undercut at the cervical portion. Down at the point there is almost no cut whatever, neither is there any at the sides. That cavity is as near as I could make it to the cavity that has the filling in it. It was filled in this manner: Soft cement was placed in the cavity, and a little crystal mat gold crowded into it. The gold does not go into the retaining-point at the cervical portion of the filling at all. It is held in entirely with cement. I would ask Dr. Perry to take out that filling and test it. Here is an excavator, and if he will say how much strength he can find there, he will satisfy me as well as illustrate this. I wish to state that I have never put a filling of this kind into the mouth, because I have not yet the confidence to do it, and I simply made this as an experiment. I did put into a cavity of this kind a filling exactly like this, and exhibited it at the meeting of the American Academy in Boston. I asked the president of the society to take it out, which he did by placing it on the table, and with a large excavator removing it, and he not only took it out, but destroyed the tooth; so I thought I would try it again, and you see the result. This specimen, I find, is a failure. It is the gold that has given way and not the cement. Whether the fault was in putting it in, or whether the cement prevented the cohesion of the gold, I cannot say. Here is an opportunity for experimenting. In placing in these fillings, we must use more gold. I have put many fillings into teeth where the retention of the gold has been entirely by the cement, but there were undercuts in the cavity, and I have never had one of those fillings fail.

The amateur photographer is abroad in the land, and he has served me a purpose. Whether it will be of service to the rest of you remains to be seen; but here is a film of celluloid, such as is used by photographers. I have found it a most admirable capping for pulps. It is very fine, and by moistening it with spirits of camphor it becomes soft, and placed over a pulp will stick to the cavity, and it makes a very nice pulp-capping. Here are a number of films of various thicknesses.

*A Member.*—Why would not that make a good matrix? Then you could see into the cavity.

*Dr. Clapp.*—It would make a good matrix for amalgams and cements, but in gutta-percha the heat might affect it. I doubt, however, whether it would have the necessary strength for gold or tin-gold. I saw suggested somewhere, and I have been trying to find an opportunity of testing it ever since, the use of aluminum. I think the color would light up the cavity and make it very valuable. Gilded or silvered copper is also used. If there is any question to be asked in regard to this filling, I shall be pleased to answer.

*Dr. Perry.*—Will you tell us the degree of dryness of the amalgam that you use, and the kind of crystal gold, and also about how soft is the oxyphosphate, and how soon is the gold put into it? Also what kind of oxyphosphate?

*Dr. Clapp.*—I use various kinds. I have had a great deal of trouble with oxyphosphates in the last year. For a long time I used Fletcher's cement, which was admirable. I think it must be eight or nine years ago that I used it quite extensively, until the acid portion of it would come in the condition of jelly, and it would soon harden so it could not be used. Then I took up Weston's cement, which I used with much satisfaction until a little more than a year ago, when that became so bad that I gave it up. I tried various other cements. Dorsen's was very good, but it did not last in the mouth. After I had used Weston's, I took up Fletcher's again, but I do not think it wears as well as Weston's when it is good. Within the last two or three months I have been using Weston's cement again, and it works much better than it did a few months ago, but still not as well as formerly. Those are about all the cements that I have used to any extent.

*A Member.*—Have you tried Paulson's?

*Dr. Clapp.*—Yes, years ago; but I had difficulty in getting it. With the last employed I had the same trouble as with Fletcher's,—the acid portion became so hard that I could not use it. I have one show filling of Paulson's cement, however: it is in the mesial surface of a superior wisdom-tooth, where it was very difficult to put anything else in. I saw the filling about eighteen months ago, and it was good, and I think it had been there nine or ten years.[1] That was a remarkable case of longevity. In using the cement with the gold, I have it as soft as very soft putty, but so it will hold its form when I put it in the cavity. I have been using for inside work Fletcher's cement more than any other, because it does not set quite as rapidly as Weston's, and it depends on how soon it does set before the rest of the filling can be completed. Usually by the time the matrix is adjusted, if amalgam be used, it can be placed in at once. It only takes three or four minutes if you are using gold. The kind of amalgam I have used more extensively than any other is Caulk's white alloy. I do not know that it is a perfect amalgam.

As to the dryness of the amalgam, I use it the same as I would to make an entire filling,—neither very dry nor very moist.

*Dr. Perry.*—Do you remove the excess of mercury by using first a portion of gold?

*Dr. Clapp.*—No; if I happen to have rather more mercury than I think is desirable, a little pressure brings the mercury to the surface, and I can scrape it off.

*Dr. Perry.*—You use the crystal gold invariably?

*Dr. Clapp.*—Formerly I used Steurer's plastic gold. Lately I have used White's crystal mat gold, although crystal gold could be used in cement where no amalgam is used equally as well as the plastic gold, and it also can be used in connection with soft amalgam, but it does not work as well. The great advantage of the plastic gold, to my mind, is that when you place an instrument on a piece of it you simply consolidate it. The instrument goes into it as your finger would go into a soft snow-ball, while the other is like putting your finger into a ball of cotton,—it pulls.

*Dr. Francis.*—In the combination of gold and amalgam, do you use gold-foil?

*Dr. Clapp.*—For making the union between the gold and the amalgam, I use a plastic gold. After the gold has thoroughly coated the amalgam the filling can be finished with any gold.

*Dr. Francis.*—Have you used gold-foil in that connection the same as plastic gold?

*Dr. Clapp.*—I have tried it, but have never made a success of it.

---

[1] I saw this filling March 23, and it was perfect. It has been in about fourteen years.

I doubt if Dr. Francis quite understands what I meant to say. It is this: after placing the amalgam into the cavity, I place the plastic gold immediately into it, and continue with the plastic gold until the amalgam is thoroughly coated with plastic gold and the filling looks like an ordinary gold filling; then any kind of cohesive gold is used.

*Dr. Francis.*—But would you not commence with gold-foil?

*Dr. Clapp.*—No; I am obliged to use the crystal or plastic gold.

*Dr. Davenport.*—Does Dr. Clapp care what proportion of cement is used? Does he use only a thin film of cement, or quite a good deal, if it be a large cavity?

*Dr. Clapp.*—I think that is immaterial, provided the cement is allowed to cover the entire portion of the cavity, with the exception of a thin circle at the margin; but I see no reason for making a large proportion of the filling gold; there is no benefit in it. We could just as well make the larger portion of the filling cement, and save time, and have a less amount of metal in the tooth, as long as we have sufficient metal to protect the cement.

*Dr. Bogue.*—Was the first specimen that Dr. Clapp passed around simply oxyphosphate and gold?

*Dr. Clapp.*—Yes.

*Dr. Bogue.*—That had only a thin film of gold. Is there only a small amount of gold in the cavity?

*Dr. Clapp.*—In this case there was considerable gold, because it was built up.

*Dr. Perry.*—You do not give the preference to Watt's crystal gold in making the union with amalgam?

*Dr. Clapp.*—No; I prefer the plastic. I have used Watt's crystal gold in only a few cases. It can be used, but it has not worked as satisfactorily in my hands.

*Dr. Perry.*—Do you prefer White's crystal mat gold?

*Dr. Clapp.*—No, I do not prefer it over Steurer's gold, except that it comes in a little more convenient form. The quality is no better than Steurer's.

*Dr. Ives.*—Since Dr. Clapp read his paper, four years ago, I have been an enthusiastic admirer and experimenter of that principle, in and out of the mouth, with a great deal of satisfaction and a large measure of success, and I feel like blessing him very often. Just now I want to speak of one case which he mentions, of a lower molar with a deep-seated cavity and good strong walls, in which he places oxyphosphate, which I consider a dangerous thing near the pulp. It is just in those cases that I would use the preparation called Fletcher's nerve-capping, which is an oxysulphate of zinc. I have been using it for many years. It is perfectly non-irritant, and hardens in a very short time. I use it invariably in all those deep-seated cavities.

One other little point: I have never yet seen a matrix for this work that in any manner approached the one he showed us at that time,—a simple piece of German silver fastened with waxed floss. You can take a burnisher and get the contour you want. I have never found any later productions that could approach it.

*The President.*—We will pass this subject, and come to the paper of the evening, by Dr. J. Adams Bishop, entitled "Injuries and Diseases of the Mouth, and their Treatment."

Dr. Bishop then read his paper, and showed some illustrations and casts which he had brought with him.

(For Dr. Bishop's paper, see page 321.)

*Dr. Bishop.*—Here we have a picture of the four-tailed bandage; the next is Gibson's, which was introduced in 1827; here is Barton's, and the next is Hamilton's, which you find in both editions. The next is in Mulin's "Modern Surgery." There he has drilled through the front of the fracture and wired it together. You see these bandages are on the outside of the face. What will you find between that and the broken bone? Let me say, for illustration's sake, Perhaps you have been to Barnum's show, and have seen the iron-jawed woman who holds her own weight by her teeth while she is being elevated thirty feet. If the muscles can hold up that one-hundred-and-fifty-pound weight, what good are those bandages when the muscles are between the external bandages and the broken bone? What good would that wire do with a muscle that can lift one hundred pounds or more?

This model represents Dr. Gunning's splint; you cannot see the white and the black line so well on the board, but I will show you some models. Here I have a model of a fractured jaw which has no support. When you put on external bandages you do not help it much, but with this interdental rubber splint you see how steadily it is supported.

The first red rubber that is being passed around is the splint that Dr. Gunning wore in his own mouth. The next two are Bellevue Hospital cases. The fourth is a private case, that of a Mr. B——, on Washington Square, and the next is the splint that Secretary Seward had in his mouth. The next is the edentulous case, and the black one is what I would make now in all cases.

Dr. Bishop also exhibited some jaw cups which Dr. Gunning considered useful for taking impressions in the country, where conveniences were not at hand.

*Dr. Carr.*—I have nothing new to say upon this subject. I have had considerable experience in the treatment of fractures of the maxillæ, and generally use for these fractures a modified Gunning splint. I must take exception to the essayist's treatment of the Barlow case. He describes the displacement as being inward and outward. In such cases I should use the wire splint invented by Dr. Hammond, of Paris, and improved by Dr. Gibson, of New York City; but in all fractures where the displacement is downward and upward, I should use a vulcanite splint,—either the modified Gunning, or the Gibson splint. An advantage of using the Gibson splint is that the patient has free use of the lower jaw.

I think the essayist is under a misapprehension when he states that fractures of the maxillæ rarely occur. Four such cases were reported in the daily papers of the past week. An explanation for the *apparently* few cases of fractured maxillæ may be given in the fact that these cases do not usually fall into the hands of the general practitioner, but are taken either to a hospital or to a dentist for treatment. If Dr. Bishop had treated the Seward case, he would have discarded all the external paraphernalia that he has shown us this evening, as being unnecessary for the treatment of such cases.

*Dr. Perry.*—It seems that Dr. Elliott saw Secretary Seward in Japan or China, and he said there was not complete recovery. We should like to hear a little more about this case.

*Dr. Bishop.*—There was some difficulty in the fracture on the right side, as I mentioned. The bicuspid tooth was very weak. The alveolus and the gum were receding, and the assassin had severed that duct and the large muscle, which made it very difficult. The duct discharged through that fracture for a long time, and made it very trying to heal. The tooth finally came away. There was union there, but you remember that Dr. Elliott saw Mr. Seward some six years after; besides, the man was a paralytic then, and he could not carry a spoon to his mouth. The conditions were very different at the time that Dr. Elliott saw the case.

*Dr. Perry.*—There was complete union, you say?

*Dr. Bishop.*—Yes; so he wrote Dr. Gunning. I saw Mr. Seward the next November, and there was nothing that indicated any such condition as Dr. Elliott wrote in his article ten years afterwards.

*Dr. Carr.*—Why do you keep the splint on from forty to sixty days?

*Dr. Bishop.*—It is not necessary in all cases; but in fractures people are very careful, and they have more confidence with the splint on. If they wish, they can remove it earlier.

*Dr. Woodward.*—I met Mr. Seward while on his tour around the world, and I happened to be in company with him when he was eating some fruit. I noticed that he held his hand on one side of his jaw when biting. I had occasion to speak to a friend of his the next day, and he informed me that his jaw was weak. He did not say that there was not a union, but it was necessary, when masticating, to hold his hand upon one side of the jaw in this way.

*Dr. Bishop.*—It was very natural for Mr. Seward to find just such a condition as your president has reported. This case which I have here is one of absorption, so that the bone was as thin, almost, as a knife-blade, and broke of itself. The splint was put on, but the man only lived about a week afterwards. In Secretary Seward's case the molar and bicuspid had been extracted, and the injury to that duct made it very difficult indeed. I remember that there was a dispute. Dr. Gunning wanted to save the bicuspid, and Medical Director Wilson said it had better come out. I remember, in the fall, when Mr. Seward visited New York with Medical Director Wilson, they came to Dr. Gunning's office, and they had some words there about having the tooth out. Mr. Seward had such confidence in Dr. Gunning, however, that he left the tooth in. After that, I do not know what happened, but when he started to go around the world, I know he was a paralytic.

*Dr. Francis.*—I would move the thanks of this Society to Dr. Clapp for his excellent practical remarks, and also to Dr. Bishop for his interesting paper.

Motions carried unanimously.

Adjourned.

JOHN I. HART, D.D.S.,
*Editor New York Odontological Society.*

